# Sclerotherapy for the Management of Seromas: A Systematic Review

**Published:** 2017-08-28

**Authors:** Aditya Sood, Vasanth S. Kotamarti, Paul J. Therattil, Edward S. Lee

**Affiliations:** ^a^The Ohio State University Medical Center, Department of Plastic and Reconstructive Surgery, Columbus, OH, USA; ^b^Rutgers New Jersey Medical School, Division of Plastic and Reconstructive Surgery, Newark, NJ, USA

**Keywords:** sclerotherapy, sclerosing, sclerosis, seroma, Morel-Lavallée

## Abstract

**Objective:** Despite improved recognition of risk factors, plastic surgeons commonly encounter seromas postoperatively and must decide upon management. Current recommendations for minimally invasive, chemical management originate from the literature on management of pneumothorax and pleural effusions. A handful of published reports have suggested the efficacy of sclerotherapy in seroma management. The aim of this study was to assess the literature on the use of sclerosants to treat subcutaneous fluid collections. **Methods:** A systematic review of the literature was performed on the PubMed, MEDLINE, and Cochrane databases for primary research articles on sclerotherapy for seroma treatment between January 1975 and January 2017. Exclusion criteria were surgical treatment, sclerotherapy for seroma prevention, hematoma, or absence of detailed documentation. Data related to seroma location, sclerosant, and resolutions were extracted. **Results:** The literature search yielded 7 relevant articles of level IV evidence and 12 case reports, with a total of 84 patients treated with sclerotherapy for persistent seromas. Slerosant included talc, tetracycline antibiotics, ethanol, polidocanol, erythromycin, OK-432, fibrin glue, and povidone-iodine. All agents achieved high rates of success. Repeat aspirations and instillations were easily performed when required. Complications, while uncommon, included pain, tightness or discomfort of the treated area, and infection. **Conclusion:** Sclerotherapy appears to be effective and safe for recurrent seromas. While a variety of sclerosing agents may be applied successfully, talc and tetracyclines remain popular choices. Because of the small scale and retrospective nature of the published literature, larger, randomized, comparative studies are necessary to assess and optimize this treatment approach.

Seromas are common complications faced by plastic surgeons.^1^^-^3 These fluid collections often cause aesthetic deformity or compression of neighboring structures. In certain circumstances, they may become super infected and present as abscesses.^1^^,^^3^^,^^4^ Additional procedures including percutaneous aspiration, drain replacement, and even returns to the operating room may be required for resolution.^1^^,^^3^

Collections of serous fluid develop through a multifactorial process. Inflammation as well as transected blood vessels and lymphatic channels contribute to accumulation of plasma, lymph, and inflammatory exudate in a surgically or traumatically created space.^1^^,^^3^^,^^5^^,^^6^ Shear forces may create or maintain the dead space, preventing apposition and adhesion of the tissue surfaces. Morel-Lavallée lesions are unique examples of subcutaneous fluid collections, specifically formed after blunt trauma, that exert shear forces separating the subcutaneous tissue from the underlying fascia.^5^^,^^7^ While seromas contain a largely acellular fluid, Morel-Lavallée lesions are filled with serosanguinous fluid and cellular debris.^7^ Allowed to persist, these postsurgical or posttraumatic fluid collections will form pseudocysts as the cavity becomes lined by fibrous tissue lacking a proper epithelium.^6^

Efforts to prevent seromas have identified several independent risk factors including elevated body mass index (BMI), large dead space, electrocautery, and time-controlled drain removal.^1^^,^^3^ Minimizing these risks through reducing dead space, maintaining lymphatic integrity, and treating high-risk patients conservatively are tenets of preventing seroma formation.^1^ Unfortunately, seromas may form nonetheless. While most serous collections resolve with conservative management, some require repeat drainage or additional therapies ranging from excision of the cavity lining to injection of a sclerosant.^5^

Sclerotherapy involves filling the seroma cavity with an irritating substance, which induces a fibrotic response to seal the dead space. Recommendations are derived from the thoracic surgery literature addressing pleural effusions.^1^^,^^3^ Commonly used substances for malignant pleural effusions include doxycycline, bleomycin, ethanol, and talc.^1^ Few published reports document the use of sclerosants to treat subcutaneous seromas. Those that exist suggest that this treatment is effective and well tolerated. However, a comprehensive, comparative analysis of the different possible options is lacking.

Several sclerosants stimulate fibrous union of the tissue surfaces by inducing inflammation. Ethanol, for example, is a common pleurodesis agent and causes sclerosis through protein coagulation and hyperosmolar cell destruction, which ultimately lead to tissue necrosis. The resulting inflammatory reaction causes fibrosis and closure of the pseudocyst.^8^ Polidocanol, an approved sclerosant for varicose veins, promotes vascular sclerosis by provoking endothelial inflammation leading to necrosis or apoptosis and ultimately thrombosis of the treated vessel.^6^ In a rabbit pleurodesis model, both erythromycin and tetracycline produced robust inflammatory responses and fibrosis.^9^ Hypotheses for the sclerosing action of doxycycline include the destruction of mesothelial cells lining the pseudocyst as well as the inhibition of fibrinolysis and the induction of fibroblast growth factors.^10^

Sclerosants that stimulate tissue adhesion without an inflammatory response have been reported as well. Fibrin glue contains fibrinogen, factor XIII, thrombin, and calcium to stimulate the final stage of the clotting cascade. The fibrin that is produced directly adheres tissue surfaces together without stimulating inflammation.^5^ Talc (hydrated magnesium silicate) is one of the most commonly used agents in the treatment of subcutaneous fluid collections but acts through a poorly defined mechanism.^3^ When used in pleurodesis, talc stimulates a fibrotic reaction involving polymporphonucleocytes as well as the cytokines interleukin-8 and basic fibroblast growth factor.^11^^,^^12^ However, in a porcine model of axillary lymph node dissection, prophylactic talc administration produced no histologic signs of active inflammation or acute inflammatory process.^4^ It has been hypothesized that in subcutaneous tissues, talc may not induce inflammation. Instead, it may increase friction between tissue surfaces and therefore reduce the effects of shear forces.^3^^,^^4^

A unique agent, OK-432, is a lyophilized mixture of a low-virulence (Su) strain of *Streptococcus pyogenes* incubated with benzylpenicillin potassium. While it performs an immunomodulatory role in the treatment of tumors, the agent may treat seromas without producing a significant inflammatory response.^13^ Instead, through stimulation of cytokine release, it may increase endothelial permeability and accelerate lymph flow.^13^ In lymphangiomas, OK-432 stimulates increases in cytokines related to granulation tissue formation and fibrotic changes.^14^

Recognizing that sclerotherapy may be an effective option for seroma treatment, we aimed to assess the published literature on subcutaneous seroma sclerotherapy and evaluate whether any substances are more effective or contribute less to patient morbidity.

## METHODS

A comprehensive search of the PubMed, MEDLINE, and Cochrane databases was conducted by the authors from January 1975 through January 2017 using the following terms alone or in combination: “sclerotherapy,” “sclerosing,” “sclerosis,” “seroma,” “Morel-Lavallée,” “sclerodhesis,” “sclerodesis,” “serodesis,” and “seromadesis.”

Prospective inclusion criteria were the use of sclerosing agents to treat postoperative or posttraumatic seromas in soft tissue and the English language. Surgical treatment of seromas, prevention of seroma formation, and hematomas, literature reviews, articles lacking detailed documentation of patients, methods, and results were excluded. In addition, articles that could not be accessed in their entirety were excluded.

Data collected included patient population information (ie, age, sex, BMI, and disease severity, duration, and stability) as well as all pertinent information on the sclerodesis procedure performed: type of anesthesia; donor site; sclerosing agent; administration method; complications, revisions, and patient satisfaction. Because of the small sample sizes and heterogeneity of the included studies, statistical analysis proved difficult and a qualitative analysis was performed alternatively.

## RESULTS

### Literature search

The primary search yielded 71 articles, of which 35 remained after initial screening and their abstracts were reviewed. Nineteen articles met our predetermined inclusion and exclusion criteria and were selected for review. There were 7 retrospective case series of level IV evidence and 12 case reports (Table 1). The earliest articles were a case series published in 1997, followed by a case report in 1999. Data from a total of 84 patients were reported, 48 of which were in case series. The remaining patients were described in case reports. Pertinent findings from this systematic review of the literature are reported in the following text.

### Patient selection

Treated patients had a mean age of 55.8 years (range, 10-85 years). The sclerosants administered included talc (6 studies, 14 patients, 16 seromas), doxycycline (3 studies, 20 patients, 20 seromas), ethanol (2 studies, 6 patients, 6 seromas), polidocanol (2 studies, 7 patients, 7 seromas), erythromycin (2 studies, 5 patients, 5 seromas), OK-432 (1 study, 1 patient, 1 seroma), fibrin glue (1 study, 1 patient, 1 seroma), rolitetracycline (1 study, 14 patients, 14 seromas), and a combination of ethanol, povidone-iodine, or doxycycline (1 study, 16 patients, 18 seromas).

### Talc

Talc was employed as a sclerosant in 6 retrospective case series or case reports to treat a total of 14 patients. The talc was applied as a dry powder (3 studies), a slurry with or without local anesthetic (3 studies), or as an aerosol (1 study).^11^^,^^12^^,^^15^^-^18

Holthouse and Chleboun^15^ applied talc aerosol to 5 seromas of the popliteal region and groin in 4 patients. Drains were placed in 3 of 5 seromas after sclerodesis. Resolution of all seromas occurred within 2 weeks, and no complications were reported.^15^

Luria et al^12^ treated 5 seromas in 4 patients located on the outer thighs and buttocks. A slurry of 5-g talc in 50-mL sterile saline was instilled into the seroma cavity, and compression bandages and drains were placed. Drains were initially connected to continuous wall suction, followed by attachment to a vacuum drainage system. Three seromas resolved within 1 to 2 weeks. In the remaining patient, who had bilateral, infected seromas prior to talc administration, 1 seroma resolved within 3 weeks. The contralateral seroma required repeat talc instillations at day 12. This patient presented 4 months postoperatively with recurrent infected seroma that was successfully managed with aspiration and antibiotics. Two patients reported asymptomatic induration of the treated areas.^12^

Lehr and Schuricht^16^ treated abdominal wall seromas in 3 patients that were refractory to previous doxycycline instillation. After cauterization of the entire seroma cavity with an argon beam coagulator, the authors applied a slurry of 4-g talc in 30 mL of bupivacaine. Abdominal binders were applied postoperatively. The seromas resolved in 2 patients by the 2- and 6-month follow-up appointments. The remaining patient had persistent fluid at 6 months, which was drained without further recurrence. No complications were reported.^16^

Catsman et al^17^ and Saeb-Parsy et al^11^ each treated 1 patient with chest wall seromas following breast surgery. In both cases, dry talc was applied, followed by compression bandages and drain placement. The patients recovered within 2 to 3 weeks, and no complications were reported.^11^^,^^17^ Metcalfe et al^18^ treated one patient with a groin wound seroma after endovascular abdominal aortic aneurysm repair. Dry talc was applied followed by a drain. The drain was discontinued within 4 days, and the wound was clean and dry by the eighth postoperative day, with no complications reported.^18^

### Tetracyclines

Tetracycline class antibiotics were used as the primary sclerosants in 4 studies to treat a total of 34 patients. The tetracyclines used included doxycycline (3 studies) and rolitetracycline (1 study).^10^^,^^19^^-^21

Bansal et al^10^ applied doxycycline to trunk, thigh, and gluteal seromas in 16 patients. Seroma cavities were instilled with 500 mg of doxycycline in 25-mL normal saline, and compression garments were applied postoperatively. Thigh lesions healed within 4 weeks, whereas lesions involving the anterior abdominal wall resolved by 8 weeks. The increased duration was associated with difficulty in applying compression bandages. One patient who was noncompliant with compression therapy had persistent fluid accumulation at 12 weeks, which resolved by the 16-week follow-up after being counseled on the importance of compression therapy. Reported complications included reduced skin mobility or tightness (11 patients), contour deformity (3 patients), and inability to run long distances following anterior thigh lesion (1 patient).^10^

Tejwani et al^19^ treated Morel-Lavallée lesions in the knees of 3 professional athletes refractory to repeated aspirations. Doxycycline was instilled in a 20-mg/mL concentration, and compressive bandages were applied postoperatively. All patients were able to resume athletic activities the following day and did not experience recurrence. Painless induration of the subcutaneous tissues was reported, however.^19^

Singh et al^20^ managed a posterior trunk seroma in a single individual with doxycycline. Thirty milliliters of doxycycline at an unspecified concentration was instilled, followed by compression bandage. Complete resolution within 1 year was reported.^20^

Widgerow et al^21^ treated 14 patients with seromas of the trunk, thighs, and other unspecified regions. The sclerosant was a mixture of 275 mg of rolitetracycline in 10- to 40-mL normal saline and 10 mL of 1% lignocaine. Abdominal seromas healed after 2 sessions of treatment 1 week apart, whereas the thighs required a mean of 4 sessions 1 week apart. The other unspecified areas healed within 2 weeks. The authors noted that excessive burning and severe discomfort were reported when local anesthetic was not included.^21^

### Ethanol

Ethanol was used as a sclerosant in 2 studies, which treated 6 seromas in 6 patients.^2^^,^^8^^,^^22^

Penaud et al^8^ treated 5 patients with 100% ethanol. Under general anesthesia, the Morel-Lavallée cavities were washed with normal saline and hydrogen peroxide, pure ethanol was instilled for six 90-second periods, and drains and compression garments were placed. Complete clinical resolution within 1 month was reported for 4 patients. The remaining patient had a persistent fluid collection one third of the original size. The authors reported a single instance of second-degree skin injury secondary to an ethanol leak.^8^

Isaacson and Stavas^22^ addressed a Morel-Lavallée lesion in the thigh of 1 patient with dehydrated alcohol. Under moderate sedation, alcohol was instilled into the cavity for 10 minutes and a drain was placed. The lesion significantly decreased in size over the following 2 weeks, with less than 5 mL per day of drainage. A repeat instillation with 5-mL alcohol for 1 hour was performed, and a drain was placed again. No complications were reported.^22^

### Polidocanol

Use of polidocanol solution or foam for sclerotherapy was reported in 2 studies, with a total of 7 patients treated.^6^^,^^23^

Moritz et al^6^ treated lower extremity seromas after varicose vein surgery in 6 patients. Polidocanol foam was applied to seroma cavities, followed by compression bandage application. Seromas resolved following a mean of 2.5 sessions. In all cases requiring repeat treatments, significant reduction in volume was achieved after the first session. No complications were reported.^6^

Laverson^23^ managed an abdominal wall seroma in 1 patient with a history of tetracycline allergy. The author administered a 1% solution of polidocanol. On the fifth postoperative day, a persistent seroma of half the original volume was aspirated without recurrence. A transient, pink discoloration of the skin was observed for 1 day.^23^

### Erythromycin

Erythromycin was used in 2 studies to treat seromas in a total of 5 patients.^24^^,^^25^

Ali-Khan et al^24^ treated gluteal and inguinal seromas in 4 patients. The authors instilled an erythromycin solution (1 g in 20-mL sterile water). One patient underwent capsulectomy prior to sclerotherapy. Three seromas resolved after a single session, whereas the remaining seroma required an additional 8 aspirations over 15 days. The authors reported 1 instance of lymphangitis within 48 hours of erythromycin administration, which resolved following oral analgesia and leg elevation.^24^

Salgado et al^25^ addressed a seroma of the hip and thigh in a single patient. After curettage of the pseudocapsule, the authors instilled 2 g of erythromycin in 200-mL normal saline and placed a drain. The drain was removed on the fourth postoperative day without any signs of recurrence or complications.^25^

### OK-432

Use of the sclerosing agent OK-432 was reported in 1 case report. Fasching and Sinzig^13^ treated a seroma overlying the sacrum by administering 0.2 mg of OK-432 in 10-mL normal saline. Leakage from the lesion ceased immediately, and fluid accumulation stopped within 4 weeks. The patient experienced 1 day of slight pain, elevated temperature, and local swelling without erythema after treatment.^13^

### Fibrin glue

Fibrin glue was used to treat a seroma of the knee by Berkoff et al.^5^ The cavity was sealed after fibrin glue administration, and the lesion resolved within 2 weeks. The authors reported no complications.^5^

### Combination sclerosants

Throckmorton et al^2^ treated 18 mastectomy site seromas in 16 patients. Initial sclerotherapy was performed by instilling 95% ethanol or dilute povidone-iodine for 20 to 30 minutes, with drains placed for postoperative management. Several patients received ethanol with doxycycline during repeat treatments. The rationale for different sclerosing agents was not reported. In addition, patients were instructed to irrigate the seroma 2 to 3 times daily at home with dilute povidone-iodine. Repeated treatments were performed if drainage was greater than 15 to 20 mL for 24 hour, if the sinogram demonstrated residual cavity size greater than 20 mL, or if the catheter was draining poorly. Fifteen of 18 seromas resolved, and the remaining 3 recurrent seromas were each treated successfully with a single aspiration. Infection was reported in a total of 7 patients, including 6 of 10 patients not receiving antibiotic prophylaxis and 1 of 5 patients receiving antibiotic prophylaxis. Five patients required hospital admission and intravenous antibiotics.^2^

## DISCUSSION

Seromas are common complications addressed by plastic surgeons.^1^^,^^3^ While most resolve with conservative management (ie, closed-suction drain, aspiration), recurrent or refractory seromas can cause discomfort and distress or present with infection.^3^^,^^4^ Morel-Lavallée lesions present with a unique etiology; however, the treatment goals remain identical to prevent the same adverse effects.^19^

Despite the diverse mechanisms of the analyzed sclerosants, high rates of success were reported for all agents, with few complications. For 10 of the 13 patients treated with talc, resolution occurred within 2 weeks. 11^,^^12^^,^^15^^-^18 Repeat aspirations or talc administration was required in 2 patients, and 1 patient was treated successfully for infected seroma recurrence with aspiration and antibiotics.^12^^,^^16^ Complete resolution was achieved in all patients receiving tetracycline antibiotics without serious complications.^10^^,^^19^^-^21 Doxycycline, the most commonly reported tetracycline in our analysis, is a widely available, effective sclerosant with a desirable safety profile. In addition, it is not associated with the severe pain and fever reported with talc, and its intrinsic antibacterial activity may prevent infection.^10^

Ethanol, polidocanol, erythromycin, OK-432, and fibrin glue likewise were applied successfully, resulting in complete resolution or significantly reduced fluid collection following a single session in most cases reported. 2^,^^5^^,^^6^^,^^8^^,^^13^^,^^22^^-^25 The higher number of sessions required for lower extremity seromas treated with polidocanol foam by Moritz et al^6^ may have been secondary to the difficult etiology (varicose veins), which was not present in the other analyzed studies. Unfortunately, only a few, small-scale studies reported the use of these sclerosants, limiting their generalizability.

Few complications were reported following sclerotherapy. One instance of second-degree skin injury following an ethanol leak highlights the need for care when handling this agent in high concentrations.^8^ No systemic complications were reported with talc administration.^11^^,^^12^^,^^15^^-^17 Instances of acute respiratory distress syndrome and systemic inflammatory response syndrome following talc pleurodesis have been attributed to systemic absorption of particles smaller than 10 µm. However, talc administered in the subcutaneous space may be less prone to systemic absorption than in the pleural cavity.^4^

Infection was an uncommon complication among the analyzed studies. The only reported infection following talc treatment was in a patient who initially presented with infected seromas. The presence of infection during initial presentation may contribute negatively to sclerotherapy outcomes through an increase in inflammatory exudate as well as the persistence of an infectious focus.^12^ Interestingly, an overall infection rate of 44% was reported in 1 study, including 60% in patients not receiving prophylactic antibiotics. Patients in this study, unlike the other studies, were instructed to irrigate the seroma cavity with dilute povidone-iodine through the drains. This issue highlights the lack of consensus regarding optimal postoperative drain management.^26^^-^27

## CONCLUSION

Despite improved recognition and management of risk factors, seromas remain a common problem addressed by plastic surgeons. Sclerotherapy is a minimally invasive method to eliminate dead space, preventing fluid accumulation. Talc, tetracyclines, and other chemical agents have been applied successfully with low complication rates. Unfortunately, because of the small scale, heterogeneity, and retrospective nature of the studies, the reproducibility and generalizability of the reported outcomes cannot be determined. Large-scale, randomized, comparative studies are needed to evaluate and compare the efficacy of these chemical agents in the treatment of seromas.

## Figures and Tables

**Table 1:** Characteristics of cited studies

First author	Year	Journal	Number of patients	Sclerosant
Widgerow^21^	1997	*South African Journal of Surgery*	14	Rolitetracycline
Laverson^23^	1999	*Plastic & Reconstructive Surgery*	1	Polidocanol
Holthouse^15^	2001	*Journal of the Royal College of Surgeons of Edinburgh*	4	Talc
Lehr^16^	2001	*JSLS Journal of the Society of Laparoendoscopic Surgeons*	3	Talc
Luria^12^	2006	*Journal of Orthopedic Trauma*	4	Talc
Saeb-Parsy^11^	2006	*The Breast Journal*	1	Talc
Fasching^13^	2007	*European Journal of Pediatric Surgery*	1	OK-432
Tejwani^19^	2007	*The American Journal of Sports Medicine*	3	Doxycycline
Throckmorton^2^	2008	*American Journal of Surgery*	16	Ethanol, povidone- iodine, doxycycline
Ali-Khan^24^	2009	*Journal of Plastic, Reconstructive & Aesthetic Surgery*	4	Erythromycin
Penaud^8^	2011	*Journal of Plastic, Reconstructive & Aesthetic Surgery*	5	Ethanol
Bansal^10^	2013	*Injury*	16	Doxycycline
Berkoff^5^	2013	*Knee Surgery, Sports Traumatology, Arthroscopy*	1	Fibrin glue
Isaacson^22^	2013	*Journal of Vascular and Interventional Radiology*	1	Ethanol
Metcalfe^18^	2013	*Annals of Vascular Surgery*	1	Talc
Moritz^6^	2013	*Phlebology*	6	Polidocanol
Catsman^17^	2016	*SpringerPlus*	1	Talc
Salgado^25^	2016	*Journal of Orthopaedic Case Reports*	1	Erythromycin
Singh^20^	2016	*BMJ Case Reports*	1	Doxycycline
